# Opinions and Use of Depth of Anaesthesia Assessment Methods: A Cross‐Sectional Survey

**DOI:** 10.1002/nop2.70801

**Published:** 2026-09-04

**Authors:** Kati Knudsen, Magnus Lindberg

**Affiliations:** ^1^ Faculty of Health and Occupational Studies, Department of Caring Sciences University of Gävle Gävle Sweden

**Keywords:** assessment, cross‐sectional survey, depth of anaesthesia, nurse anaesthetist, nursing process

## Abstract

**Aims:**

To capture nurse anaesthetists' opinions on the use of depth of anaesthesia assessment methods and describe when and how nurse anaesthetists find such technical devices useful in their clinical practice.

**Design:**

Cross‐sectional.

**Methods:**

Exploratory web‐based survey among (*n* = 79) nurse anaesthetists who were members of the Swedish National Association for Anaesthesia and Intensive Care.

**Results:**

In total, 25 nurse anaesthetists estimated that they used anaesthesia depth monitoring in up to every fourth case, whereas 20 reported applying monitoring in up to every second patient. Situations in which nurse anaesthetists would use depth of anaesthesia monitoring were during total intravenous anaesthesia, and in previous instances of awareness. There was a divergence of opinion among the nurse anaesthetists regarding whether judgement of clinical signs was equivalent or superior to the use of anaesthesia depth monitoring.

**Conclusion:**

Based on the survey results, there are indications that knowledge regarding depth of anaesthesia assessment methods needs to be enhanced.

**Implications for the Profession:**

Anaesthesia depth monitoring is regarded as important in specific clinical situations, rather than as a routine component of all general anaesthesia. Variations in nurse anaesthetists' opinions on the value of clinical signs compared with technological monitoring indicate that these approaches may be best understood as complementary rather than mutually exclusive methods of assessing anaesthetic depth. Furthermore, the findings highlight the need for ongoing professional education and discussion regarding the appropriate indications, interpretation, and application of anaesthesia depth monitoring in nurse anaesthesia practice.

**Impact:**

The findings suggest that anaesthesia depth monitoring is not routinely used by responding nurse anaesthetists. The results further indicate that nurse anaesthetists' opinion and experiences toward this technology vary, highlighting the importance of considering these factors when developing structured guidelines for clinical practice.

**Reporting Method:**

Strobe guidelines were followed.

**Patient or Public Contribution:**

No patient or public contribution.

## 1 Introduction

The nurse anaesthesia practice integrates advanced nursing and anaesthetic competence to provide safe, person‐centred care throughout the perioperative period. Central to the role are continuous assessment, vigilance, airway management, prevention of complications, and the ability to act in rapidly changing clinical situations. However, nurse anaesthesia extends beyond technical proficiency: it encompasses establishing trust during brief patient encounters, recognising vulnerability, protecting dignity, advocating for the patient and contributing to effective interprofessional collaboration. Research demonstrates that these dimensions are integral to anaesthesia nursing and that high‐quality practice involves continuously assessing and responding to the patient as a whole person (Lekens et al. 2023; Pann et al. 2025).

Thus, nurse anaesthetists play a crucial role in ensuring patient safety during surgery (Engdahl et al. 2026), particularly when patients are asleep or in an unconscious state. An essential component of safeguarding patient well‐being is the accurate monitoring of anaesthetic depth. By observing clinical parameters, such as blood pressure, heart rate, pupil responses, eye reflexes, and tear secretion, and remaining vigilant to anticipate potential complications, nurse anaesthetists can respond rapidly to unexpected events (Nilsson and Jaensson 2016).

Technical devices, such as the Bispectral Index (BIS) (Mathur et al. 2023; Lewis et al. 2019) and Entropy (Chhabra et al. 2016), provide objective methods for monitoring the depth of anaesthesia. These tools aid in minimising the risks of under‐ or oversedation, ensuring the patient maintains an appropriate level of general anaesthesia, and reducing the likelihood of intraoperative awareness (Ford and Simmons 2023). Despite the potential benefits of these monitoring devices, their routine integration into clinical practice by nurse anaesthetists remains unclear. However, a recent Norwegian study indicated that six out of ten nurse anaesthetists utilised depth of anaesthesia monitoring for over 50% of their patients undergoing general anaesthesia (Aasheim et al. 2024).

## 2 Background

Systematic reviews have demonstrated that BIS‐guided general anaesthesia offers several advantages over reliance on clinical parameters. These include a reduced incidence of intraoperative awareness, earlier extubation, improved postoperative recovery, for instance regarding orientation in time and place, and consequently earlier discharge from both the operating room and the post‐anaesthesia care unit. Nevertheless, the robustness of these findings is limited, as the studies reviewed were characterised by low‐certainty evidence (Oliveira et al. 2017; Lewis et al. 2019).

It is well‐known that overly deep anaesthesia can cause haemodynamic instability, and that overly light anaesthesia involves an increased risk of awareness or recall during general anaesthesia. The BIS target range for sufficient depth of general anaesthesia has been suggested to be between 40 and 60. However, BIS monitoring should always be adapted to the context, the patient's characteristics and vital signs, and if a muscle relaxant is used. BIS should not be used as the sole measure of anaesthesia (Zhang et al. 2011; Abdelmalak and Doyle 2010; Mathur et al. 2023).

Technical and non‐technical skills, along with critical thinking, are crucial for nurse anaesthetists to administer anaesthetics safely (Olin et al. 2023; Lyk‐Jensen et al. 2014). Caring for a patient during surgery is thus challenging, and technical devices such as BIS and/or Entropy can support the nurse anaesthetist in ensuring patient safety. Although previous research has demonstrated several benefits of using depth of anaesthesia monitoring during general anaesthesia, it remains unclear in which situations nurse anaesthetists find these technical devices useful in their clinical practice. As nurse anaesthetists' opinions and experiences can influence their actions in clinical practice, it would be valuable to explore how they apply and perceive different methods for assessing the depth of anaesthesia.

## 3 The Study

### 3.1 Aims

The purpose of this study was to capture nurse anaesthetists' opinions on the use of depth of anaesthesia assessment methods and describe when and how nurse anaesthetists find such technical devices useful in their clinical practice.

## 4 Methods

### 4.1 Design

Cross‐sectional exploratory survey.

### 4.2 Instrument With Validity and Reliability

A web‐based survey on opinions on and use of depth of anaesthesia monitors was conducted between 10 June 2024 and 30 September 2024. The survey questions were adapted from a questionnaire previously used for the same purpose among Australian anaesthesiologists (Ben‐Menachem and Zalcberg 2014). Permission to translate, adapt, and reuse the questionnaire was obtained from the primary author of the Australian study (e‐mail correspondence Erez Ben‐Menachem). A forward–backward translation was conducted in accordance with the established method described by Beaton et al. (2000) and the final version was validated by a specialist nurse anaesthetist. The adaptations were both contextual, ensuring that the framing of the questions aligned with the organisation of the Swedish healthcare system, and specific to the role of nurse anaesthetics. The phrasing of the questions was tailored to reflect the professional responsibilities and scope of practice of nurse anaesthetists, ensuring that participants could directly relate to the content and recognise its relevance to their practice. Additionally, the response format for some of the original questions was modified from a simple yes/no format to a four‐point Likert scale ranging from ‘does not agree at all’ to ‘fully agrees’, which was more suitable for measuring opinions. These Likert scale responses was coded 1–4 in the dataset. The survey items and response options underwent a thorough review to assess their meaningfulness, clarity and appropriateness for respondents, and were subsequently approved by representative of the Swedish National Association for Anaesthesia and Intensive Care.

### 4.3 Sampling and Recruitment

The setting for this study was established within the membership registry of the Swedish National Association for Anaesthesia and Intensive Care. However, the study was specifically limited to the subset of members who were actively engaged in anaesthesia care. The association is a non‐profit professional organisation, founded in 1962, and aimed at specialist nurses and students within anaesthesia, intensive care, and post‐operative care. Its vision is to promote quality and safety, education, lifelong learning, and research and development grounded in scientific evidence and proven experience. All current nurse anaesthetist members were eligible to participate in the study. No inclusion criterion was applied.

An administrator with authorised access to all members' contact details (e‐mail addresses) as part of their role assisted the researchers in distributing invitations for participation. This procedure ensured that the researchers did not process members' personal data. Using the membership system, potential participants were sent information about the study, including an invitation to participate and a link to the survey. Those who voluntarily chose to participate confirmed within the web survey that they had received and read the study information and had the opportunity to ask questions and get responses from the researchers prior to participation. No reminders were sent, as it was not possible to track respondents who had already completed the survey, and the intention was to avoid inconveniencing those who had already participated.

### 4.4 Data Analysis

All descriptive and most inferential statistics were calculated in IBM SPSS Statistics for Windows (Version 29.0.2.0. Armonk, NY: IBM Corp) using a complete case analysis approach. The data are presented in tables or figures, with rates expressed as percentages or actual numbers. Kendall's tau‐b test was applied to exploratory assess the association between nurse anaesthetists' years of professional experience and the self‐reported frequency of anaesthesia depth monitoring use. The Fisher–Freeman–Halton (FFH) exact test was applied to exploratory assess the association between nurse anaesthetists' years of professional experience and their self‐reported opinions on the use of anaesthesia depth monitoring. The FFH test was also employed to examine the association between situations in which nurse anaesthetists would employ depth of anaesthesia monitoring and the self‐reported frequency of anaesthesia depth monitoring. To account for multiple comparisons, resulting *p*‐values were adjusted using the Holm–Bonferroni method, with significance set at *p* < 0.05. This analysis was performed using R software (version 4.5.1. Vienna, Austria: R Foundation for Statistical Computing).

### 4.5 Ethical Approval

All procedures in the study were conducted in accordance with the most recent update of the Declaration of Helsinki (Brazil 2013). The study protocol was approved by the Swedish Ethical Review Authority, registration number 2024‐00293‐01 on 27 February 2024.

## 5 Results

### 5.1 Subjects

Seventy‐nine nurse anaesthetists working in either a university hospital (*n* = 32, 40.5%), county hospital (*n* = 29, 36.7%), regional hospital (*n* = 13, 16.5%), or private hospital (*n* = 5, 6.3%) participated in the study. Most were women (*n* = 51, 64.6%) and active in the southern parts of Sweden. They had on average worked 13.0 years (SD 9.0, median 11) as nurse anaesthetists and were on average 47.1 years old (SD 8.9, median 46). According to the completed web surveys, anaesthesia depth monitors were absent at the workplaces of 11 nurse anaesthetists, whereas 65 reported having access to BIS and only two had access to Entropy. In addition, two respondents stated that they also had access to Sedline. One in five nurse anaesthetists reported that they had experienced an instance of awareness in an anaesthetised patient.

### 5.2 Use of Depth of Anaesthesia Monitoring

Twenty‐two nurse anaesthetists (28.2%) reported never using anaesthesia depth monitoring as a tool during anaesthesia, whereas two (2.6%) stated that they always did. In total, 25 nurse anaesthetists (32%) estimated that they used anaesthesia depth monitoring in up to every fourth case, whereas 20 (25.6%) reported applying monitoring in up to every second patient. The remaining nurse anaesthetists (*n* = 9, 11.6%) used anaesthesia depth monitoring for most of their patients, but not always. No statistically significant association was identified between nurse anaesthetists' years of professional experience and the self‐reported frequency of anaesthesia depth monitoring (Kendall's tau‐b test = 0.093, *p* = 0.261).

Two thirds of the respondents indicated that they would use anaesthesia depth monitoring during total intravenous anaesthesia (TIVA) and 60% would use it if the patient had previously had an instance of awareness. Table 1 presents the various situations for which nurse anaesthetists were asked to indicate whether or not they would use depth of anaesthesia monitoring. Only certain procedures under general anaesthesia, specifically trauma surgery and caesarean section, showed a significant association with self‐reported frequency of anaesthesia depth monitoring (Table 1). The effect size calculated using Cramer's V was 0.67 and 0.53, respectively.

**TABLE 1:** Description of situations in which a nurse anaesthetist would use depth of anaesthesia monitoring (*n* = 79), with associations to self‐reported utilisation assessed via the Fisher–Freeman–Halton exact test.

Situation	Yes/No, *n*	Fisher–Freeman–Halton exact test raw *p* (Holm–Bonferroni adjusted *p*)
Under total intravenous anaesthesia (TIVA) (*n* = 79)	53/26	0.016 (0.112)
When the patient has previously had instances of awareness (*n* = 79)	47/32	0.355 (0.894)
Under general anaesthesia with muscle relaxant (*n* = 79)	42/37	0.133 (0.532)
Under general anaesthesia with remifentanil and anaesthetic gas (*n* = 79)	23/56	0.037 (0.222)
Under general anaesthesia (*n* = 79)	17/62	0.038 (0.222)
Under general anaesthesia during trauma surgery (*n* = 79)	17/62	0.003 (0.024)^a^
Under general anaesthesia during a caesarean section (*n* = 79)	14/65	0.002 (0.018)^a^
Under general anaesthesia during thoracic surgery (*n* = 79)	14/65	0.298 (0.894)
Not in any of the above situations (*n* = 79)	2/77	0.919 (0.919)

^a^ Fisher–Freeman–Halton exact test raw *p*‐values have been Holm–Bonferroni adjusted; significance is indicated at the 0.05 level.

### 5.3 Opinions on the Use of Anaesthesia Depth Monitoring

**FIGURE 1:**
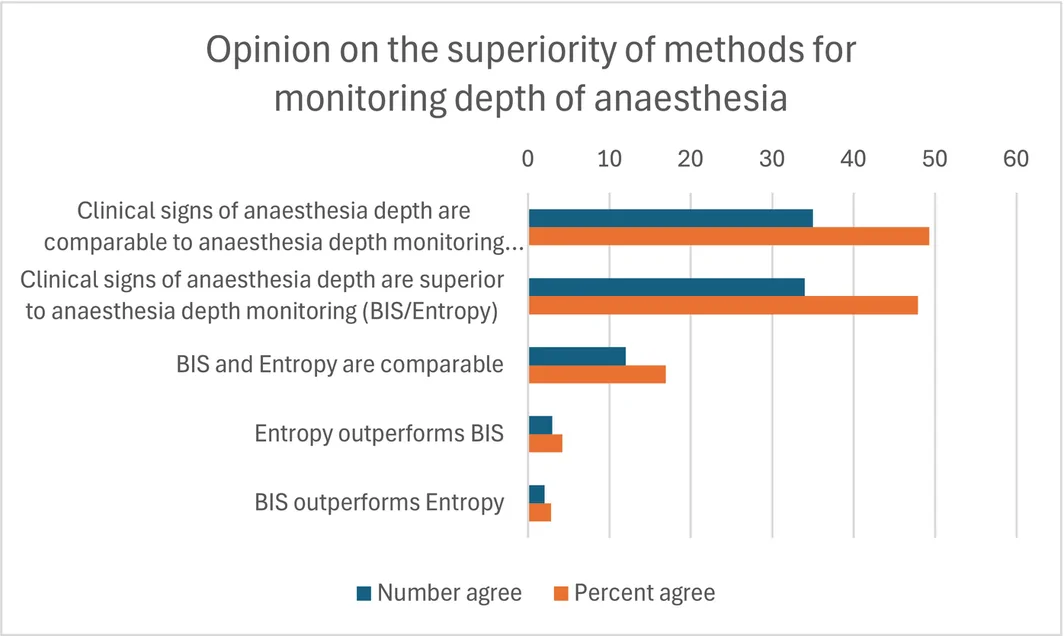
Opinion on clinical signs versus depth of anaesthesia monitoring, given in actual numbers (*n* = 71).

There was a divergence of opinion among the participating nurse anaesthetists regarding whether judgement of clinical signs was equivalent or superior to the use of anaesthesia depth monitoring (Figure 1). Approximately every second nurse anaesthetist had the opinion that assessment of blood pressure, heart rate, pupil responses, eye reflexes, and tear secretion was equivalent to anaesthesia depth monitors. Almost as many stated that clinical signs were superior to monitoring. The Fisher–Freeman–Halton exact test (=34.980, raw *p* = 0.031, *n* = 71) indicated a statistically significant association between years of professional experience and the opinion that clinical signs of anaesthetic depth are equivalent to anaesthesia depth monitoring. The effect size calculated using Cramer's V was 0.73. No statistically significant associations were identified for the remaining opinions (Figure 1).

**FIGURE 2:**
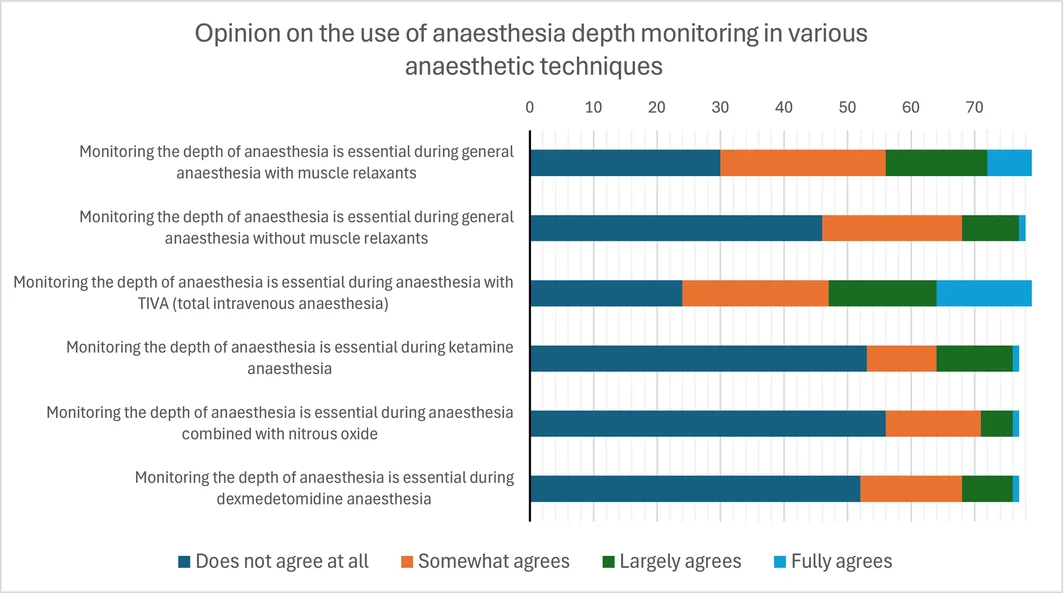
Opinion on monitoring in various anaesthesia procedures, given in actual numbers (*n* = 79).

Nearly 8 out of 10 responding nurse anaesthetists were of the opinion that it was necessary to apply anaesthesia depth monitoring during TIVA and 7 out of 10 agreed to at least some extent that monitoring should be used during general anaesthesia with a muscle relaxant. Monitoring the depth of anaesthesia was not considered essential during the use of ketamine anaesthetics or nitrous oxide (Figure 2).

**FIGURE 3:**
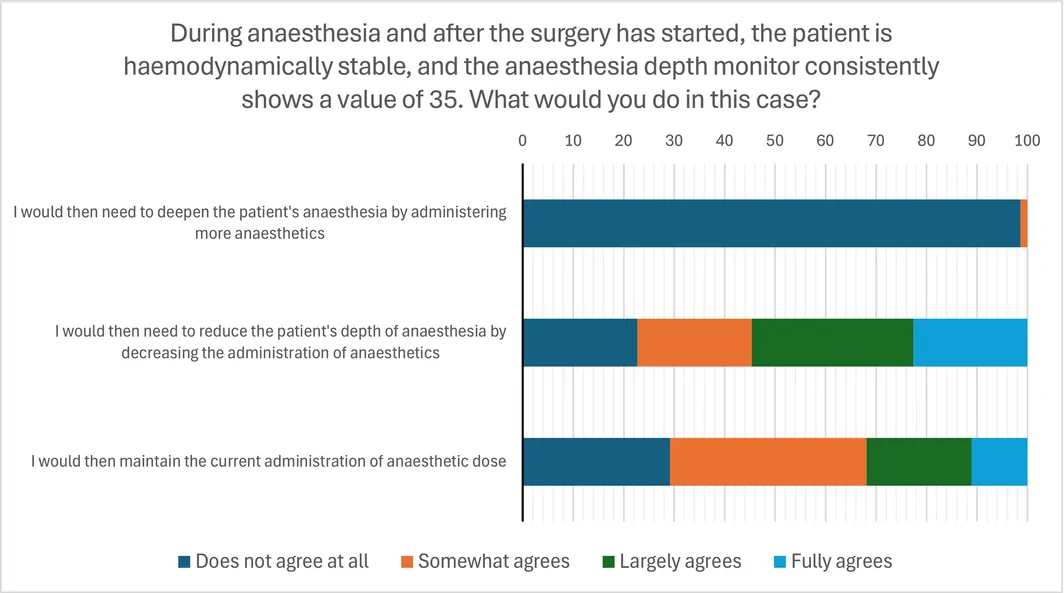
Opinion on anaesthetic dose adjustments at anaesthesia depth monitoring index 35. Likert scale responses reported in percentages (*n* = 72).

**FIGURE 4:**
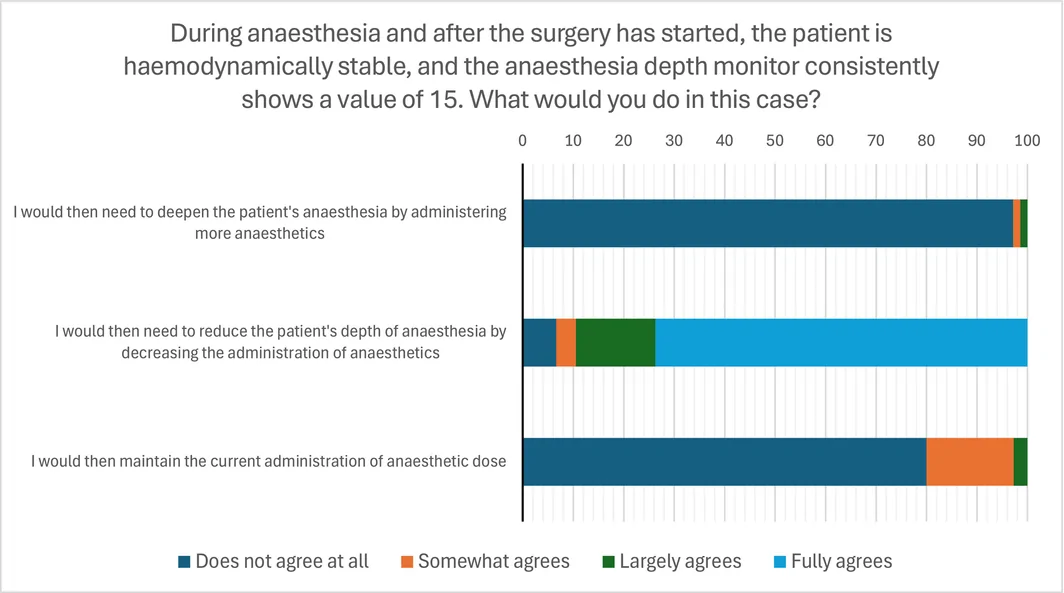
Opinion on anaesthetic dose adjustments at anaesthesia depth monitoring index 15. Likert scale responses reported in percentages (*n* = 74).

**FIGURE 5:**
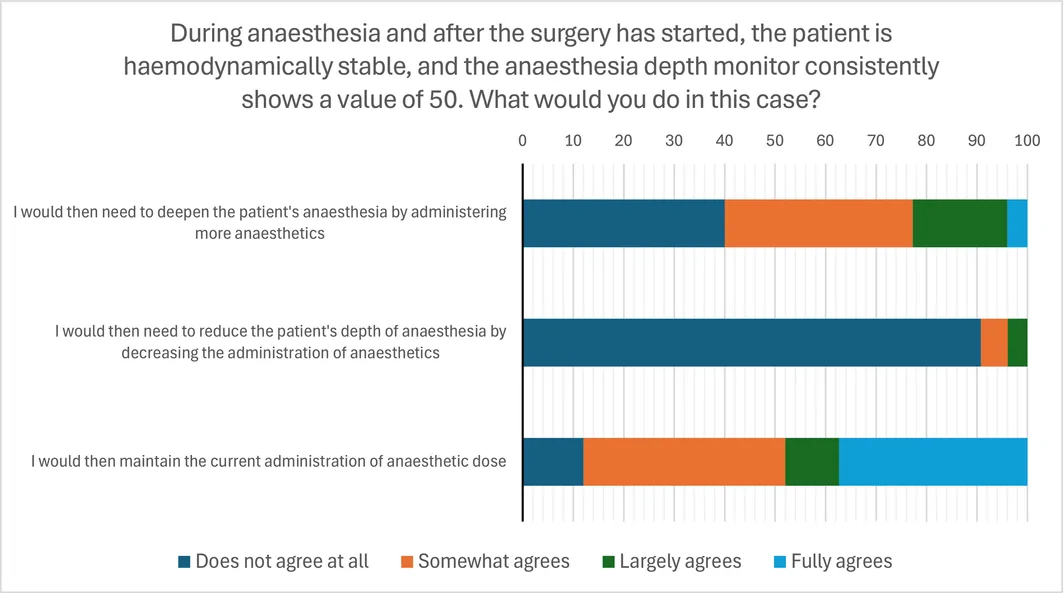
Opinion on anaesthetic dose adjustments at anaesthesia depth monitoring index 50. Likert scale responses reported in percentages (*n* = 75).

When asked about what anaesthetic adjustments they would make in case of certain monitoring readings, almost all nurse anaesthetists responded that they would maintain or decrease the administration of anaesthetics if the value was 35. However, a few would deepen the anaesthesia (Figure 3). In a situation where the value was 15, 9 out of 10 nurse anaesthetists would decrease the administration of anaesthetics, whereas almost 2 out of 10 would maintain the level of administration. Two nurse anaesthetists (2.8%) stated that they would administer more anaesthetics to a patient with a value of 15 (Figure 4). A quarter of the nurse anaesthetists were of the opinion that a longer period of low anaesthesia depth during surgery did not increase the risk of serious postoperative complications for the patient. In the situation when the anaesthesia monitoring showed a value of 50, 6 out of 10 nurse anaesthetists would administer more anaesthetics to the patient, whereas 4 out of 10 nurse anaesthetists fully agreed with maintaining current administration (Figure 5).

The nurse anaesthetists stated that anaesthesia depth monitoring was valuable for preventing both over‐ and underdosing of anaesthetics during general anaesthesia. However, the majority did not believe that such monitoring facilitated a controlled and safe awakening or reduced the incidence of postoperative dysfunction and complications (Table 2).

**TABLE 2:** Opinions on what depth of anaesthesia monitoring enables, based on statements given. Likert scale responses reported in actual numbers.

Use of anaesthesia depth monitoring provides the opportunity	Does not agree at all	Somewhat agrees	Largely agrees	Fully agrees
To better control general anaesthesia (*n* = 78)	2	31	32	13
To avoid overdose of anaesthetic agents (*n* = 78)	1	24	37	16
To avoid underdosing of anaesthetic agents (*n* = 78)	3	32	32	11
To ensure successful awakening from anaesthesia (*n* = 78)	21	38	14	5
To reduce the risk of postoperative cognitive dysfunction (*n* = 75)	6	42	22	5
To reduce costs associated with anaesthesia (*n* = 77)	12	38	19	8
To mitigate climate change (*n* = 77)	9	38	19	11
To decrease the number of postoperative complications (*n* = 75)	8	39	24	4
To make the patient more hemodynamically stable intraoperatively (*n* = 78)	11	38	24	5

## 6 Discussion

This study aimed to capture nurse anaesthetists' opinions on use of anaesthesia depth assessment methods in clinical practice. The web survey responses showed that most of the nurse anaesthetists had access to BIS, whereas only two had access to Entropy in their departments. The current literature has shown that BIS and/or Entropy might be valuable tools for monitoring depth of anaesthesia and can provide an additional parameter to consider in safeguarding patients' wellbeing during anaesthesia. However, these monitoring devices should be interpreted alongside conventional clinical signs such as blood pressure, heart rate, and pupil responses (Ford and Simmons 2023).

Although there are benefits to using BIS and/or Entropy during general anaesthesia, our survey responses show that such monitoring is not frequently used by nurse anaesthetists. These findings are consistent with those of an earlier study, which found that anaesthesia professionals had a sceptical view of using depth of anaesthesia devices, and such monitoring was used in only 50% of the hospitals (Jildenstål et al. 2014). Surprisingly, little has changed in clinical practice, based on both previously published results and our survey findings. However, the majority of nurse anaesthetists self‐reported that they used anaesthesia depth monitoring during TIVA or when a muscle relaxant was used, and if the patient had previously had an instance of awareness. Despite this, only trauma surgery and caesarean section performed under general anaesthesia demonstrated a statistically significant association with self‐reported utilisation. This finding suggests that, although depth of anaesthesia monitoring is conceptually endorsed in clinical situations traditionally regarded as high risk, its consistent use may be driven more by procedural context and perceived clinical demands than by anaesthetic technique per se. Nevertheless, as utilisation was self‐reported, these findings should be interpreted with caution owing to the potential for reporting bias.

UK guidelines and recommendations for standard monitoring during general anaesthesia and recovery have been published. These guidelines state that anaesthesia depth monitoring should be used as a minimum standard when administering TIVA with neuromuscular blocking (Klein et al. 2021). However, there is an ongoing debate regarding the practical utility of anaesthesia depth monitoring in clinical practice (McCulloch and Sanders 2023; Myles 2023). In the absence of national guidelines and recommendations for standard monitoring of anaesthetic depth, it is not surprising that the responding Swedish nurse anaesthetists in this survey considered clinical parameters to be superior to anaesthesia depth devices.

The responding nurse anaesthetists were of the opinion that a longer period with a low anaesthetic depth value did not increase the risk of serious postoperative complications for their patients. This finding is somewhat remarkable, suggesting that they do not consider the patients' postoperative well‐being. In addition, most of the responding nurse anaesthetists did not agree that anaesthesia depth monitoring facilitated a controlled and safe awakening or reduced the risk of postoperative cognitive dysfunction. In contrast, several studies have shown benefits of using anaesthesia depth monitoring to reduce time to extubation, orientation in time and place, and discharge from both the operating room and the post‐anaesthetic care unit (Oliveira et al. 2017; Lewis et al. 2019).

### 6.1 Limitations

This study had several limitations. One limitation is the use of a web‐based survey, as this tends to result in low response rates, especially in nurse populations (L'Ecuyer et al. 2023). Because the total population of potential respondents is unknown, the true denominator for calculating incidence cannot be established. As a result, the incidences presented here reflect only the opinions of those who chose to participate and may not accurately represent the broader population of nurse anaesthetists in Sweden. Consequently, there is a risk of selection and non‐response bias inherent in this study. Moreover, a low response rate limits the generalisability of the findings, as the results may not represent the population (in this case, nurse anaesthetists working in Sweden). However, the results can be considered indicative of the prevailing opinion at Swedish anaesthesia units. The present study may also be prone to non‐response bias due to the low response rate; those with a particular interest in anaesthesia depth monitoring devices may have been more likely to respond. These concerns are consistent with previous research reports from other countries (Aasheim et al. 2024; Ben‐Menachem and Zalcberg 2014). The fact that an administrator from the association distributed the invitation to participate in the study means that the researchers lacked oversight regarding the exact number of members who actually received the web survey. This prevents calculation of an accurate response rate. However, this procedure contributed to a higher degree of privacy protection for the association's members, which had been unconditionally promised in the ethical review.

### 6.2 Implications for Nursing Practice

The findings suggest that anaesthesia depth monitoring is perceived as particularly relevant in selected clinical situations, rather than as a routine component of all general anaesthesia. Depth of anaesthesia monitoring was considered important during total intravenous anaesthesia and for patients with a history of awareness. Furthermore, the association between monitoring frequency and specific procedures, for example during trauma surgery and caesarean section, suggests that decisions regarding the use of depth of anaesthesia monitoring may be influenced by procedure‐specific characteristics and perceived clinical risk.

At the same time, the variation in nurse anaesthetists' opinions regarding the equivalence or superiority of clinical signs highlights the importance of recognising clinical assessment and technological monitoring as potentially complementary rather than mutually exclusive approaches. The findings also indicate a need for continued professional education and discussion regarding the appropriate indications, interpretation, and use of anaesthesia depth monitoring in nurse anaesthesia practice.

### 6.3 Recommendations for Further Research

Larger studies are needed to fully determine opinions on and any benefits of using depth of anaesthesia monitoring in routine care, especially for frail patients who undergo anaesthesia. Further research is needed to gain a deeper understanding of when and how nurse anaesthetists find this technology useful in their clinical practice. One way forward may be to develop and implement structured clinical guidelines and recommendations to ensure safe patient care.

## 7 Conclusion

Based on the survey results, there are indications that knowledge regarding depth of anaesthesia assessment methods needs to be enhanced.

## Author Contributions

**Kati Knudsen:** conceptualization, methodology, visualization, writing – original draft, formal analysis, project administration, funding acquisition. **Magnus Lindberg:** conceptualization, visualization, writing – review and editing, formal analysis, methodology.

## Funding

The authors have nothing to report.

## Disclosure

The authors have checked to make sure that our submission conforms as applicable to the journal's statistical guidelines. The manuscript does not include advanced statistical analyses that would require consultation with a qualified statistician. The authors are well‐versed in the applied methodology and can ensure its correct implementation without the need for statistical consultancy. The authors agree to take responsibility for ensuring that the choice of statistical approach is appropriate and is conducted and interpreted correctly as a condition to submit to the Journal.

## Ethics Statement

The study was approved by the Swedish Ethical Review Authority (Diary number: 2024–00293‐01).

## Conflicts of Interest

The authors declare no conflicts of interest.

## Data Availability

The data that support the findings of this study are available on request from the corresponding author. The data are not publicly available due to privacy or ethical restrictions.
